# Developing an Integrated, Brief Biobehavioral HIV Prevention Intervention for High-Risk Drug Users in Treatment: The Process and Outcome of Formative Research

**DOI:** 10.3389/fimmu.2017.00561

**Published:** 2017-05-11

**Authors:** Roman Shrestha, Frederick Altice, Pramila Karki, Michael Copenhaver

**Affiliations:** ^1^Department of Community Medicine and Health Care, University of Connecticut Health Center, Farmington, CT, USA; ^2^Institute for Collaboration on Health, Intervention, and Policy, University of Connecticut, Storrs, CT, USA; ^3^AIDS Program, Department of Internal Medicine, Section of Infectious Diseases, Yale University School of Medicine, New Haven, CT, USA; ^4^Department of Allied Health Sciences, University of Connecticut, Storrs, CT, USA

**Keywords:** HIV prevention, preexposure prophylaxis, neurocognitive impairment, people who use drugs, combination approach, biobehavioral community-friendly health recovery program

## Abstract

To date, HIV prevention efforts have largely relied on singular strategies (e.g., behavioral or biomedical approaches alone) with modest HIV risk-reduction outcomes for people who use drugs (PWUD), many of whom experience a wide range of neurocognitive impairments (NCI). We report on the process and outcome of our formative research aimed at developing an integrated biobehavioral approach that incorporates innovative strategies to address the HIV prevention and cognitive needs of high-risk PWUD in drug treatment. Our formative work involved first adapting an evidence-based behavioral intervention—guided by the Assessment–Decision–Administration–Production–Topical experts–Integration–Training–Testing model—and then combining the behavioral intervention with an evidence-based biomedical intervention for implementation among the target population. This process involved eliciting data through structured focus groups (FGs) with key stakeholders—members of the target population (*n* = 20) and treatment providers (*n* = 10). Analysis of FG data followed a thematic analysis approach utilizing several qualitative data analysis techniques, including inductive analysis and cross-case analysis. Based on all information, we integrated the adapted community-friendly health recovery program—a brief evidence-based HIV prevention behavioral intervention—with the evidence-based biomedical component [i.e., preexposure prophylaxis (PrEP)], an approach that incorporates innovative strategies to accommodate individuals with NCI. This combination approach—now called the biobehavioral community-friendly health recovery program—is designed to address HIV-related risk behaviors and PrEP uptake and adherence as experienced by many PWUD in treatment. This study provides a complete example of the process of selecting, adapting, and integrating the evidence-based interventions—taking into account both empirical evidence and input from target population members and target organization stakeholders. The resultant brief evidence-based biobehavioral approach could significantly advance primary prevention science by cost-effectively optimizing PrEP adherence and HIV risk reduction within common drug treatment settings.

## Introduction

Even with numerous HIV prevention interventions, HIV incidence in the US has not decreased in the past 15 years (1). People who use drugs (PWUD) remain a priority population as they represent a critical conduit for new HIV infections, which occur through preventable HIV risk behaviors (2–6). An increasing number of evidence-based behavioral strategies to prevent HIV have been developed for high-risk populations, including PWUD (7), which have rightfully remained central to HIV risk reduction and medication adherence. Despite the implementation of extensive behavioral interventions over the past decades, these strategies have not been sufficiently effective to show comparable outcomes among PWUD (8, 9). Therefore, there is an urgent need to incorporate innovative strategies that take into consideration the specific risk reduction needs of this population to improve existing HIV prevention approaches.

Significant advances in biomedical HIV research [e.g., preexposure prophylaxis (PrEP)] have been made over the past few years. The recent availability of PrEP—the daily self-administration of antiretroviral medication for primary HIV prevention—provides an unprecedented opportunity to curtail the HIV epidemic. Evidence from recent PrEP trials has demonstrated its safety and efficacy in significantly reducing the risk of HIV acquisition for those at substantial risk of acquiring HIV infection, including PWUD (10–14). Consequently, the Centers for Disease Control and Prevention (CDC) recommends PrEP in PWUD and provides clinical practice guidelines for the use of PrEP for HIV prevention (15).

Despite unequivocal evidence supporting PrEP in the US, its scale-up has been gradual overall (16), and nearly absent among PWUD. One of the primary barriers to wide-scale usage of PrEP is the requirement for near perfect adherence to the daily self-administration of PrEP. Recent trials clearly establish that the success of PrEP is dependent on behavioral factors affecting PrEP uptake and medication adherence. Importantly, these trials maximized the efficacy of PrEP by incorporating behavioral approaches that motivate clients to initiate it and then adhere to it consistently, modify their risky behaviors [e.g., prevent other sexually transmitted infections (STIs)], and help them to acquire and retain the knowledge and skills necessary to support behavior change (10–14). Therefore, experts are now calling for combination approaches to primary HIV prevention that comprises both biomedical and behavioral components for optimizing health outcomes (17–19).

Recently, accumulating evidence has demonstrated that a disproportionate percentage of PWUD (>30%) experience a wide range of cognitive deficits in various domains—such as executive function, attention, memory, new learning, information-processing speed, and visual–spatial perception (20–25)—that have significant impact on HIV risk behaviors as well as HIV risk-reduction intervention outcomes (9, 25–29). For example, deficits in executive function influence rational decision-making, which may impede individuals from making safer sexual choices. Similarly, slowed information-processing function may prevent the timely, appropriate consideration of risk variables during decision-making situations (30). In addition to chronic drug use, several cognitive risk factors, including opioid substitution therapy and comorbidities (20–22, 31–33), tend to worsen neurocognitive impairment (NCI) symptoms of PWUD to the extent that it may be disruptive to their participation in treatment services, including decreased treatment engagement (34–36), poor treatment retention (37, 38), and poor treatment outcomes (30, 33, 34, 39–42). Therefore, successful behavioral engagement of PWUD in biomedical prevention, such as PrEP uptake, continuation in PrEP program, and optimal PrEP adherence, may be complicated by the cooccurrence of NCI. The potentially disruptive impact of NCI must therefore be addressed when designing contemporary combination prevention packages targeting PWUD.

The aim of this study was to integrate an evidence-based behavioral approach with an evidence-based biomedical approach. Furthermore, we incorporated innovative strategies in the integrated biobehavioral intervention to optimally address PrEP adherence and HIV risk reduction needs of high-risk PWUD within common drug treatment settings [e.g., methadone maintenance program (MMP)]. The first aim was to adapt an evidence-based behavioral intervention by adding specific content to foster PrEP adherence and treatment engagement and incorporating strategies to accommodate individuals’ NCI. The second aim was to integrate the resultant, adapted behavioral intervention with the evidence-based biomedical approach (i.e., PrEP) to form a combination HIV prevention approach. The process and outcomes of our formative research, including the resulting integrated intervention, are outlined below.

## Materials and Methods

We prepared to develop an integrated biobehavioral approach to optimally address HIV prevention needs of high-risk PWUD by conducting formative research that first involved adapting an evidence-based behavioral intervention. We then combined the adapted behavioral intervention and the evidence-based biomedical intervention (i.e., PrEP) for optimal implementation among the target population.

### Adapting an Evidence-Based Behavioral Intervention

We used the *Assessment–Decision–Administration–Production–Topical experts–Integration–Training–Testing* (*ADAPT-ITT*) (43) model as a guide for adapting an evidence-based behavioral intervention with the goal of optimally designing it for implementation among our target population of high-risk PWUD. The ADAPT-ITT model, which is designed to help adapt existing evidence-based interventions (EBIs), consists of eight sequential steps that include *Assessment, Decision, Administration, Production, Topical Experts, Integration*, and *Training*. In this study, we used the first six steps of the ADAPT-ITT framework (see Table 1), as follows.

**Table 1:** **Applying the Assessment–Decision–Administration–Production–Topical experts–Integration–Training–Testing model to adapt the community-friendly health recovery program (CHRP) intervention for implementation among high-risk people who use drugs (PWUD)**.

Phase	Methodology
1. Assessment	Conducted focus groups (FGs) with members of the target population (i.e., high-risk PWUD) and organizational key stakeholders (i.e., treatment providers) to determine the specific needs of the target population Decisions regarding the characteristics of intervention (e.g., content, format, placement, delivery) were made to inform the adaptation of the behavioral intervention Analyzed results of formative evaluations
2. Decision	Decided to adapt the CHRP intervention defined as an evidence-based behavioral intervention by the SAMHSA
3. Administration	Theater testing was conducted during the FGs with members of the target population to examine attitudes toward the format and content of the intervention and to receive feedback and recommendation for improving acceptability of the intervention
4. Production	Revised the existing CHRP intervention based on the results of the previous phases Created the first draft of the adapted CHRP intervention while maintaining fidelity to the core elements and theory
5. Topical experts	Sought feedback from content experts on the first draft of the adapted EBI and the flow and content of the manual
6. Integration	Integrated topical experts’ feedback to create the final draft of the intervention Drew upon Wiley’s framework to develop a culturally sensitive intervention approach *[We combined the final, adapted CHRP intervention with the evidence-based biomedical intervention (PrEP)—now called CHRP-BB—designed to address HIV risk reduction and PrEP adherence challenges common among PWUD]*

**Next steps to be completed**	

7. Training	Train research assistants to assist with implementation of the biobehavioral community-friendly health recovery program (CHRP-BB) intervention, participant recruitment, and data collection during the testing of the CHRP-BB intervention
8. Testing	Administer the CHRP-BB intervention among 40 high-risk PWUD in methadone program Examine feasibility, acceptability, and preliminary efficacy of an integrated CHRP-BB intervention for adherence to PrEP and HIV risk reduction among high-risk PWUD

#### Assessment Phase

This phase involved collecting data from members of the target population (i.e., high-risk PWUD in methadone program) and organizational key stakeholders (i.e., treatment providers) using focus group (FG) sessions to determine specific needs of the target population. The objective of conducting FGs was to guide the adaptation process of the evidence-based behavioral intervention, in terms of determining (a) intervention content (i.e., specific content areas of the original intervention modules to include/exclude, emphasize/abbreviate), (b) delivery modality (i.e., group vs. individual), and (c) intervention session duration (i.e., length/timing). We focused on the key characteristics of the HIV transmission risk and NCI profile and medication adherence issues of the target population, interest in PrEP, and ways to optimize intervention content, format, and delivery in terms of accommodating cognitive deficits.

#### Decision Phase

This phase involved using results from the FG sessions to inform selection of an existing behavioral intervention targeting high-risk PWUD in a drug treatment setting. Our goal was to select an EBI that was most relevant to the target population—high-risk PWUD in a treatment context. As such, we reviewed the available EBIs and associated published reports to identify EBIs that seemed most appropriate.

#### Administration Phase

This phase involved pre-testing methodology, known as theater testing, to adapt an EBI with our target audience. FG sessions were used to examine attitudes regarding the format and content of the intervention and to collect feedback and recommendations for improving the acceptability of the intervention among members of the target audience.

#### Production Phase

This phase drew upon the results of the previous phases and involved carefully adapting content from the original EBI as well as creating a first draft of the adapted behavioral intervention. The aim was to produce a successfully adapted behavioral intervention for our target population, while maintaining fidelity to the core elements and behavioral theory.

#### Topical Experts Phase

This phase involved collecting feedback from content experts on the first draft of the intervention manual. Content experts were identified in several key domains: HIV prevention, substance abuse, PrEP as HIV prevention, and high-risk PWUD. We identified experts based on a needs assessment of our target population (phase 1). Experts were presented a draft of the adapted behavioral intervention to provide feedback and recommendations for further refinement.

#### Integration Phase

In this phase, we integrated feedback from topical experts into the adapted intervention in preparation for pilot testing. Importantly, in order to develop a culturally sensitive intervention approach, we drew on Wiley’s framework that includes accommodation, incorporation, and adaptation (44).

### Integrating a Biomedical Approach with the Adapted Evidence-Based Behavioral Intervention

Additionally, we integrated the adapted version of the behavioral intervention with the evidence-based biomedical intervention (i.e., PrEP) to optimally address the HIV prevention needs of high-risk PWUD in drug treatment. In order to assist with the integration process, the FGs also covered issues pertaining to participants’ attitude toward PrEP use, implementation of PrEP in community-based drug treatment facilities, such as MMP, the target population’s preferences (e.g., PrEP use), and logistical issues involved in the implementation of PrEP (e.g., cost, resources, and time). The key factors that we considered were (a) perceived relevance of PrEP use to overall health care and health-related quality of life among the target population, (b) participants’ likelihood of adhering to PrEP, (c) how to make PrEP optimally available to the target population, and (d) what approach would be least disruptive to the existing workflow in the clinic.

### FG Participants and Procedures

A convenience sample of participants was recruited *via* flyers, word-of-mouth, and direct referral from counselors at the APT Foundation, Connecticut’s largest methadone clinic. Potential participants were screened in-person in a private room or by phone using a screening form. Individuals who met inclusion criteria, and who were willing to participate, were provided a description of the study and invited to provide informed consent, followed by the FG. All participants were reimbursed for the time and effort needed to participate in the FG. The study protocol was approved by the Institutional Review Board at the University of Connecticut and received board approval from the APT Foundation Inc.

Between May and June 2016, we conducted structured FG sessions with members of the target population (*n* = 20) and with treatment providers (*n* = 10). Members of the target population were individuals who shared key characteristics with our target population (i.e., HIV-negative, enrolled in MMP, and presence of drug- and/or sex-related HIV risk behaviors). The characteristics of participants were as follows: female (55%), African-American (25%), age range 28–59 years (mean age = 42), participating in drug treatment (100%), and recent history of drug-related (70%) and sex-related (95%) risk behaviors. Treatment providers from a multidisciplinary team at the clinic and drug treatment facility were invited to participate based on the degree to which they assisted in the HIV-related care of the target population. Our objective was to interview treatment providers with a range of expertise assisting target population in the region. Employment characteristics of treatment provider participants were as follows: infectious disease nurses (30%), addiction counselors (30%), HIV prevention counselor (10%), physicians (20%), and administrator (10%). The demographic characteristics of treatment provider participants were as follows: female (60%), White (60%), and age range 34–65 years (mean age = 49) (see Table 2).

**Table 2:** **Characteristics of all interview participants**.

	Target population (*n* = 20)	Treatment provider (*n* = 10)
Age (years)	Range: 28–59 (mean: 42)	34–65 (mean = 49)
Gender	Female: 11 (55%)	6 (60%)
Ethnicity	African-American: 5 (25%)	African-American: 4 (40%)
	White: 13 (65%)	White: 6 (60%)
	Others: 2 (10%)	
Enrolled in drug treatment	20 (100%)	–
HIV transmission risk behaviors	Drug-related: 14 (70%) Sex-related: 19 (95%)	–
Employment characteristics	–	Infectious disease nurses: 3 (30%)
		Addiction counselors: 3 (30%)
		HIV prevention counselor: 1 (10%)
		Physicians: 2 (20%)
		Administrator: 1 (10%)

All FG sessions were structured around a set of carefully predetermined interview guides as used in prior studies (45–47) (see Tables 3 and 4). These guides were brief, structured but relatively open-ended to encourage free-flowing discussion. Participants would ask each other or to the facilitators follow-up questions. The FG sessions were led through an open discussion based on the FG guides by two trained graduate-level facilitators. A total of six FG sessions were conducted and each session lasted between 45 min and 1 h. An average of five participants, with a range of four to six, per session were included in each FG session, which was consistent with the figures quoted in the FG methods literature (48–50). All FG sessions were audiotaped with participants’ permission and were transcribed verbatim. A trained doctoral-level researcher conducted the resulting content analyses.

**Table 3:** **Structured interview instrument for collecting data from the target population participants**.

Items	Questions
1	What are the health problems or concerns that are the most important to you right now? What do you worry about the most?
2	Have you ever participated in an HIV education program or HIV prevention groups that covered drug use and sexual risk behaviors? What was the most helpful about it? What could have been improved?
3	What types of information or skills should we consider in creating a better HIV prevention program for people in health care settings or addiction treatment?
4	When you participate in group or individual counseling sessions while in treatment, do you ever have difficulty remembering details later or concentrating on what is covered? Please describe some examples…
5	What are some ways that HIV prevention material could be presented so that you could be better able to concentrate, learn, or remember details, for example (provide examples of tools—hands-on/review chunks of material/multimodal presentation/interactions with peers, etc.)?
6	In your daily life, what helps you remember things best like appointment times, when to take medications, for example? Seeing or hearing information? Using “reminders”? Practicing certain routines (e.g., taking meds before bed)?
7	What types of memory aids (e.g., phone, alarms, calendar, etc.) have you used to help you remember to do certain tasks (e.g., meet with your counselor, take medication, attend doctor’s appointment, etc.)
8	What are some other strategies that could help?
9	What you heard about the medication PrEP? (Describe the basics of what it is and how it works, if applicable). Who should take it and why?
10	If APT Foundation—like other programs—makes PrEP widely available, would you be interested in trying it?
11	Since PrEP has to be taken properly for it to work, what are the best ways you can suggest for reminding people to take it?
12	What do you think would be the greatest pros and cons of using PrEP as part of a brief (~4 group session—describe community-friendly health recovery program) HIV prevention program during treatment?
13	What other suggestions/comments can you provide?

**Table 4:** **Structured interview instrument for collecting data from the treatment providers**.

Items	Questions
1	What do you think is your patients’ level of understanding about HIV transmission risk?
2	What types of HIV risk behaviors do you perceive in your patients?
3	What do you think patients practice risky behaviors? What kinds of situations seem the most common?
4	What level of neurocognitive functioning do you see among most clients in the program?
5	What proportion would you guess have cognitive impairments that might impact their participation in treatment?
6	(If common), what strategies have you used to accommodate those patients?
7	What do you think is the role of cognitive impairment may play in their HIV risk behavior? What about their treatment engagement and outcomes?
8	What strategies do you think could help your clients to better engage in individual or group HIV prevention sessions, remember content from session, and remember to apply content?
9	What specific strategies or tools do you think would be practical/useful for incorporating into our HIV prevention program (describe community-friendly health recovery program) to accommodate individuals with cognitive impairment?
10	What do you know about PrEP? (describe the basics if they are unfamiliar)
11	How would you feel about offering/prescribing PrEP to your clients in the context of HIV prevention and treatment?
12	How interested/willing do you think your clients would be about PrEP?
13	What are some of the challenges for delivering PrEP in this treatment setting?
14	What do you think are the greatest potential barriers to and facilitators of PrEP use among clients?
15	What concerns you the most about the use of PrEP among your clients (e.g., side effects, adherence, etc.)?
16	What other suggestions should we consider?

### Data Analysis

We used Atlas.ti software to facilitate management and analysis of FG data (51). Data from the target population and treatment provider FGs were analyzed independently. Data analysis followed a thematic analysis approach, applying several qualitative data analysis procedures (e.g., inductive analysis, cross-case analysis). In the initial inductive analysis, emergent themes and patterns were identified directly in the transcripts. The coding process was completed for each FG transcript and a master list of themes was compiled to reflect overarching themes. Two research team members met regularly to become acquainted with participant narratives, to contextualize differences, to build consensus, and to cross-case analysis decisions of emerging themes. Interrater agreement was calculated by a third member of the research team, which was 95% or greater for each FG. Primary themes derived from the FG data were used to retain, update, and revise the contents from the original behavioral intervention to inform the integrated biobehavioral intervention.

## Results

### Adaptation of an Evidence-Based Behavioral Intervention

The adaptation process of the behavioral intervention followed the general principles of the ADAPT-ITT model (see Table 1), which are detailed below.

#### Assessment Phase

In order to conduct a needs assessment and to determine what would be the most relevant intervention for our target population (i.e., high-risk PWUD in treatment), we collected information from members of the target population and treatment providers using the FG sessions. Overall, the results from the FGs (both target population and treatment providers) identified the following key themes and subthemes regarding our population of high-risk PWUD for inclusion in the adapted EBI:

(a) Appropriate for the behavioral component to retain original content of the CHRP intervention related to both drug- and sex-related HIV risk reduction.
(b) Intervention should cover content specific to PrEP:
  - Basics about PrEP;
  - Potential motivation driving PrEP use (e.g., *pros and cons* of achieving high levels of adherence to PrEP);
  - Problem solving (e.g., improving strategies for identifying and overcoming obstacles to adherence);
  - Facilitators to PrEP adherence (e.g., learning memory aids for improving adherence);
  - Enhancing decision-making related to PrEP;
  - Overcoming stigma related to being on PrEP.
(c) Greater emphasis on certain strategies or tools to accommodate participants with NCI in order to help them to better concentrate, learn, and remember details:
  - Multimodal style of presenting material (e.g., oral, visual, skill-building modalities);
  - Use of simple language and frequent review of materials;
  - Assessment with immediate feedback (e.g., before and after quizzes, awarding small prizes relevant to the sessions);
  - Use of memory aids (e.g., workbook, text messaging);
  - Behavioral learning (e.g., contingency management);
  - Learning by doing (e.g., role-play, in-session short activities).
(d) Appropriate to retain the original characteristics of the intervention:
  - Format (e.g., a manual-guided intervention strategy comprises four 60-min sessions);
  - Delivery (e.g., weekly group sessions).

The results of FGs with the target population and the treatment providers are reported below.

#### FG Interviews with Target Population

##### Participation in HIV Prevention Groups

Most participants reported that they had previously participated in some form of HIV education program, particularly in a drug treatment setting, in a correctional unit, or in a rehabilitation center. Participants were asked to comment on the types of information or skills that we should consider in creating a better program in health-care settings or addiction treatment. Common responses included the following:

“Teach them responsibility and accountability…maybe now change your risky behavior.” “Basically, awareness… a lot of people only talk about contracting it through sex but there’re a lot of other ways you can get it.”

##### Strategies to Accommodate Individuals’ NCI

The majority of participants reported that they have some cognitive deficit, particularly memory and learning disabilities, which prevents them from remembering intervention content later or concentrating during sessions. Participants provided suggestions regarding possible ways HIV prevention material could be presented so that people with NCI could be better able to focus, learn, or remember details. Participants primarily recommended the use of simple language and repetition of materials, contingency management, assessment with immediate feedback, structure and consistency, use of memory aids, and multimodal presentation of material:

“You can catch people’s attention using different ways. Watching… speaking… interacting with them.” “I think what’s more helpful will be demonstrations that we do ourselves in addition to what you [group facilitator] do.” “They need to make it simpler.” “Reiterating.” ”Give them some test to help them remember after the session…and hand out something to award them if they did well.”

When questioned about what helps them remember things best (e.g., appointment times, taking medication), most participants reported the use of external memory aids, such as their phone, memory book, telephone/text reminder, alarms, and social support.

“My clinician sends me text a day before my appointment.” “I will put it in my phone. When it’s time, my phone will go off.” “I write on a piece of paper and stick on something or put it in my cell phone.” “Have family help out. People near you care for you. You need that support from your network.” “Reminder calls.”

##### Knowledge and Awareness about PrEP

With regards to PrEP, almost none of the participants had ever heard of PrEP (95%) before participating in the study. Participants who had heard of PrEP knew that it was for individuals who engage in risky behaviors and its use would stop them from being infected with HIV. Participants were concerned that others in the community may also be unaware of PrEP, and highlighted the importance of getting the information out and advocating for its uptake:

“It [PrEP] is out in the market and we don’t even know there’s a medication that prevents us from getting HIV.” “Why don’t they [counselors] bring this up during group sessions?”

##### Attitudes toward PrEP

While most participants expressed favorable attitudes toward the use of PrEP, a few expressed concerns about the complexities related to its use. The primary concerns participants raised about offering PrEP to individuals in drug treatment setting included (i) encouraging increased risky behavior, (ii) medication cost, (iii) side effects, (iv) interaction with methadone, (v) burden of daily medication, and (vi) stigma.

The most frequently mentioned concern about PrEP was the likelihood for increased risky behavior. Participants discussed issues related to the potential reduction in condom use, needle sharing, and worries related to inadvertently increasing other STIs:

“I’d be worried that by them taking it [PrEP], they’ll feel too much like superman…so they put themselves in more risky situations.” “It [PrEP] gives somebody to be not careful. If they know that they can still do this [engage in risky behaviors] and not get it [HIV], that’s going to make this [STIs] go through the roof.”

As participants were asked about the acceptability of taking PrEP, some brought up cost as one of the major barriers. They were unwilling or unable to pay for PrEP medication and were worried about insurance not paying for the cost associated with PrEP, as exemplified:

“Does insurance cover it? If there’s a copay or something on it, many people are not paying out of pocket for it.” “Obviously, I’m living on the street and don’t have a lot of money.”

Some participants expressed concerns about the potential side effects of PrEP on their health and highlighted the importance of increasing awareness about alternative approaches to HIV prevention. While, some participants were worried about the possible interaction between methadone and PrEP.

“Why would you want to mess with medication like that? You never know what side-effects you will get.” “I heard it somewhere that some HIV drugs wear off the effect of methadone.”

In general, participants thought that it would be hard for most people in drug treatment program, who are actively using drug, to take a pill consistently on a daily basis. They believed that the use of illicit drugs or cognitive deficits (e.g., memory problems) may create significant barriers to PrEP adherence:

Taking it [PrEP] on a daily basis will be a problem…because most of them [PWUD] are not responsible enough to take it [PrEP] every single day.

A few participants shared that HIV stigma is prominent in their communities and, thus, would not go to HIV clinic to get PrEP medication because of stigma related to HIV. They noted that they would avoid HIV clinics due to the potential embarrassment it could cause, as indicated:

Once I step into a HIV clinic to get it [PrEP], people will start thinking that I’ve got the virus [HIV]. I’d rather not take it.

##### Facilitators of PrEP Uptake and Maintenance

The majority of participants indicated that they would use external memory aids, such as cell phone, calendar, post-it notes, and pill container, while some suggested they would make use of social support network (e.g., friends, family members) to facilitate adherence to PrEP medication:

“I put an alarm in my phone. That’s the only way I remember.” “Put it in a daily pill container.” “My therapist reminds me to take medication. I could do the same to take PrEP.”

#### FG Interviews with Treatment Providers

##### Patients’ Engagement in Risky Behaviors

All providers tended to agree that their clients practice higher level of HIV risk behaviors, including both sharing of injection equipment and condomless sex. Some of the treatment providers indicated that these risk behaviors were prevalent mostly among most-at-risk populations, including men who have sex with men (MSM) and people who inject drug. When asked why they believed their patients continue to engage in risky behaviors, example of the common response included:

I think it’s about the inability to see forward. This will feel good now and they don’t have the intellectual or emotional capacity to see the future.

##### Patients’ Level of NCI and Its Consequences

Treatment providers mentioned that they serve a mix of clients (10–50%) with varying degrees of NCI, which affect their ability to understand, process, and retain information and skills provided during treatment. This deficit in their cognitive functioning may be due to HIV infection, other infections, chronic drug use, or aging. Almost all providers agreed that the presence of cognitive deficit among patients negatively affects their engagement in HIV risk behaviors, treatment engagement, and treatment outcomes.

There is more chance that they will engage in risky behaviors. I’ve had clients who forget to take their meds or go see their doctors even after reminding them multiple times.

When asked about some possible strategies to use to accommodate patients’ NCI, common response included:

It has to be constant, constant, constant…repetitive, repetitive, repetitive, and has to be multiple mediums…maybe one group could be video, one group a talking group, maybe one group a writing group …with before and after quizzes.

##### Knowledge and Awareness about PrEP

When asked about how familiar they were with PrEP, the majority of the providers reported that they were familiar with it and were able to describe its basics; however, very few (10%) reported having read the CDC’s clinical practice guidelines on PrEP. Surprisingly, addiction counselors, with whom the high-risk PWUD in drug treatment is mostly likely to meet on a regular basis, had relatively little awareness of PrEP.

“To be honest, I don’t know about it. You guys can educate us more about it…so that we can discuss that with the clients and the clients can follow up with you.” “I know very little, not enough, not nearly as much as I should have.”

##### Treatment Providers’ Attitudes toward PrEP

The majority of the providers agreed that the most-at-risk populations, including partners of HIV-infected individuals, sex workers, MSM, people with unsafe sexual behaviors, high-risk drug users, who are engaged in risk behaviors would make an appropriate PrEP candidate. Interestingly, a few providers indicated that they would be hesitant to offer PrEP to individuals who are cognitively impaired or have a history of substance abuse.

It’s a great idea…but I don’t think I’d treat someone who is cognitively impaired with PrEP. I doubt they’ll adhere to it [PrEP].

##### Primary Concerns Raised about PrEP during FG Sessions

Six primary themes emerged that may have implications for setting clinical protocols and informing future programs regarding PrEP: (i) PrEP acceptability, (ii) medication cost, (iii) possible increase in STIs, (iii) adherence, (iv) side effects, (v) stigma, and (vi) administrative logistics.

Although many providers tended to agree that their clients would be interested in being on PrEP, particularly if they are engaging in risky behaviors, some providers shared their concerns about the potential acceptability of PrEP among their clients.

I think for a lot of them it would be scary…But, if it’s promoted in the right way, I think people will feel comfortable about it.

When asked about some of the challenges for delivering PrEP in a treatment setting, many providers brought up issues related to medication cost as the most important one.

“I’m not sure about the insurance coverage…but it’ll definitely make a huge difference.” “With our clients, number one challenge can be insurance coverage.”

Many providers were worried that widespread PrEP use could be associated with increase in clients’ engagement in risky behaviors (i.e., engaging in risk compensation) and STIs, which could potentially increase their overall risk for acquiring HIV.

I think that people have this new freedom that they’re finding with PrEP and they may be engaging in risky behaviors still…because they feel as though the worst thing that can happen is protected against.

In addition, providers raised concerns about adherence to PrEP, particularly among individuals with mental health diagnoses or ongoing substance abuse issues.

“Adherence would concern me the most, especially someone who is actively relapsing.” “I’d be really concerned about how individuals will remember to take this med every single day.”

Some providers mentioned that they would be worried about the potential side effects associated with the use of PrEP, whereas one provider highlighted the issue about stigma that may be attached to PrEP use.

They may not want to take it stigma wise. I mean…if people see me taking it, in a second, they’ll know I’ve had risk behaviors.

#### Decision Phase

Based on the findings from the first phase and the assessment approaches used in prior studies (45–47), we concluded that the CHRP (52) would be an ideal fit for HIV prevention targeting high-risk PWUD in MMP. The CHRP is a brief, evidence-based behavioral HIV risk-reduction intervention designed for high-risk PWUD participating in community-based drug treatment settings. It is a theory-based, manual-guided intervention strategy comprised of four 50-min group sessions that address sex- and drug-related HIV risk behaviors among high-risk PWUD enrolled in MMP. The sessions are provided by two trained facilitators who deliver intervention content using cognitive remediation strategies (e.g., presenting material visually, verbally, and experientially) designed to accommodate the mild-to-moderate cognitive difficulties that are common among this population (52).

#### Administration Phase

There was universally high acceptance of the behavioral intervention among members of the target population in all FG sessions. Treatment providers also reported similar willingness to toward the behavioral intervention and no organizational barriers were identified to the successful integration of the intervention within existing programs at the research site. When asked to identify the total number and duration of the intervention sessions they thought would be feasible, the majority of the FG participants agreed that four 60-min group sessions would be feasible. This is consistent with the administration time of the original CHRP intervention. In terms of the implementation of the intervention, most participants felt that reminding participants participating in the group sessions would improve participant engagement.

#### Production Phase

Next, we developed the first draft of the adapted CHRP intervention. The changes made to the original intervention incorporated elements based on the needs of high-risk PWUD in treatment identified in Phases 1–3. We incorporated only the necessary modifications in terms of content, style, and process to the original CHRP intervention (52) as indicated in the previous phases. We also updated the quality assurance and process measures from the original intervention to reflect the adaptation.

#### Topical Experts Phase

The expert review resulted in a number of improvements to the first draft of the adapted intervention. In addition to language and format changes recommended, there was also a recommendation that we change the name of the adapted intervention from the “CHRP-NCI” to the “CHRP-BB.” Experts suggested that having “NCI” in the intervention name would potentially send a negative message to participants who may not perceive themselves to be cognitively impaired. Thus, it was recommended to included “biobehavioral” in the new name [i.e., biobehavioral community-friendly health recovery program (CHRP-BB)], as this would more accurately represent the integrated biobehavioral approach. Additionally, they suggested that handouts for each group session should be provided to participants as needed.

#### Integration Phase

Feedback from topical experts was integrated into the second draft of the adapted EBI. Based on experts’ suggestions about the need for handout materials for each session, we developed materials to be given to participants at the end of each session. We also made the suggested language and format changes and changed the name of the intervention to “CHRP-BB.”

In order for the adapted CHRP intervention to be culturally appropriate, we drew on Wiley’s framework, which includes accommodation, incorporation, and adaptation as the three initial courses of action (44). *Accommodation* required having a better understanding of the communicative styles and literacy level of PWUD in treatment and to account for such factors in the adaptation process. Because our research team has extensive experience in the development, adaptation, and evaluation of innovative HIV prevention approaches targeting high-risk PWUD, we were readily able to engage in this course of action throughout the intervention adaptation process. *Incorporation* required becoming familiar with community practices and to incorporate them in the adapted intervention. *Adaptation* involved facilitating the intervention development process with the philosophy of tailored approach to promotion of information, motivation, and behavioral skills to reduce risk behaviors and to improve medication adherence among high-risk PWUD in treatment (44).

### Integrating PrEP into the Adapted CHRP Intervention

Our results also showed an almost unanimous positive attitude about offering PrEP along with the behavioral intervention at drug treatment clinics (i.e., MMPs) and the clinics’ potential role in increasing adherence to PrEP among their clients.

#### FG Interviews with Target Population

When asked if they would be interested in trying PrEP as a way to prevent HIV, the majority of the participants, but not all, reported that they would take PrEP themselves. A few participants reported that they did not engage in risky behavior and were therefore less interested in taking PrEP. They were, however, willing to be on PrEP if their engagement in risk behavior changes in the future. Additionally, many participants believed that it is important for people who engage in risky behaviors to be on PrEP.

I think that it [PrEP] is good and should be available for the ones that need it. And if someone’s engaging in risk behavior, they should definitely take it. It doesn’t only benefit that person, it will benefit people around. When you’re an addict, you’re not thinking straight. So, I mean, when you’re not thinking with clear head and you have been on PrEP as a fall back. So for those kind of people, it’s good to have.

The convenience of the dispensing venue was identified as an important facilitator to potential PrEP uptake and maintenance. Since all of the participants were enrolled at the drug treatment clinic (i.e., MMP) to get medicated on a daily basis, they pointed out that dispensing PrEP along with methadone at the MMP may increase adherence among this high-risk group.

“Give it to them with their methadone. Because they are not going to forget to come here.” “I think the best thing will be to give them with methadone because we are always going to make sure we get methadone. Because we will get sick if we don’t take it [methadone]. And as long as we can get it in the medication window, we’re not going to forget to take it.” “I don’t care what other people think. I would definitely take it with methadone. That doesn’t bother me.”

Some participants, however, highlighted the importance of maintaining privacy while dispensing PrEP in a drug treatment setting, to ensure that they are not labeled as engaging on risky behaviors:

Some people may be embarrassed to take the medicine [PrEP] while in line for methadone. Other people will know that that person has risky behavior.

Almost all participants welcomed the idea of a combination approach to the prevention of HIV that comprises both a biomedical (i.e., use of PrEP) and a behavioral intervention:

“I would say yeah, offer it. It’s good that you guys are educating, you know. One con, I see is that people will feel invincible taking it [PrEP]…but if you’re educating them, I think that will help them to understand the entire picture.” “I think that’s a clever idea…offer pamphlets, stuff like that, to educate people on it…so that they know what they are taking and what it’s doing to your body, how it works, how it blocks it [HIV], pros and cons.” “I love the idea, man. There’re a lot of rumors out there. You need to educate these people…and having PrEP as a back-up is great. If you forget to use condom when you’re high, you’re still safe.”

#### FG Interviews with Treatment Providers

Providers were asked how they felt about offering PrEP to clients in the context of HIV prevention and treatment. Almost all providers felt positively about PrEP and indicated that it should be made available to curtail the HIV epidemic.

“I think it’s fabulous. Why should people get infected [with HIV] if they don’t have to?” “I think it’s great. Our goal is to prevent HIV, right? If it [PrEP] is like 90% effective, then why not?” “I’ll be 100% for it. I’ve seen what HIV has done to people, especially at later stages. Honestly, prevention, prevention, prevention. We do a lot of reaction and not a lot of proactive prevention.” “I feel very comfortable recommending PrEP. I always mention it to our new patient [who are HIV-infected] as options for their friends and their Partners if their partners are still negative or have yet to be tested.”

The majority of the providers believed that offering PrEP along with methadone in a community-based drug treatment setting (i.e., APT Foundation) would help to facilitate monitoring and clients’ adherence to PrEP.

“I think it’s an amazing idea to provide PrEP in drug treatment clinic. I think it’s awesome, in that, at least you can watch them take it and you know it’s in their system…because if they have to come here already for methadone why don’t you just add the pill and…boom and you don’t have to worry about them taking it on their own, which may not happen.” “Since they are already hooked up with the methadone clinic, may be…the clinic can dispense medication. I think it’ll be really easy to monitor whether or not they took PrEP.”

In terms of strategic placement of PrEP for high-risk PWUD, almost all providers welcomed the idea of offering PrEP in drug treatment settings along with a behavioral intervention and believed that the administrative staffs would be receptive to this idea.

“Administration will be open to offering every service we could possibly offer to the clients. I see this [offering PrEP along with the CHRP intervention] as one more great thing and APT’s arsenal of community help, community treatment.” “They [administrative people] are all for the research and encourage us to participate in this sort of things.”

Based on the results of this study and available research, we integrated the final, adapted CHRP intervention with the evidence-based biomedical intervention (i.e., PrEP) to form a brief biobehavioral HIV prevention intervention—now referred as the CHRP-BB. This combination HIV prevention approach, designed to address HIV risk reduction and PrEP adherence challenges common among PWUD, also incorporates strategies to address NCI.

As part of the combination approach (CHRPP-BB intervention), the target population (i.e., high-risk PWUD in drug treatment) will receive a comprehensive package of prevention services, including HIV testing, diagnosis and treatment of diagnosed STIs, methadone treatment, prescription of PrEP, and four 1-h group sessions that focus on a range of relevant topics pertaining to reducing sex- and drug-related HIV risk behaviors and specific content intended to foster adherence to PrEP with strategies carefully incorporated to accommodate participants’ cognitive impairment. The group sessions include (see Table 5): (a) making the most of PrEP as an active health manager, (b) reducing drug risk and taking PrEP, (c) PrEP adherence and sex risk-reduction strategies, and (d) negotiating partner support for HIV prevention. Equally important, we sought to incorporate only the necessary modifications in terms of content, style, and process to the original CHRP intervention (52) as indicated by the information gleaned from the structured FG sessions.

**Table 5:** **Overview of the biobehavioral community-friendly health recovery program intervention sessions**.

Session topics	Topics taught
1. Making the most of PrEP as an active health manager	Actively participating in health care, Improving skills for partnering with health care providers; Improving skills for partnering with health care provider; Understanding PrEP; *pros/cons* of taking PrEP; Building PrEP adherence skills.
2. Reducing drug risk and taking PrEP	Identifying drug-related HIV risks; Learning about proper needle cleaning; Managing drug cravings; Reducing the use of drugs while on PrEP.
3. PrEP adherence and sex risk reduction strategies	Identifying sex-related HIV risks; Learning about latex products and their correct use; Use of latex protection while on PrEP.
4. Negotiating partner support for HIV prevention	Negotiating use of latex; Communicating about PrEP and sex and drug-related HIV risk.

## Discussion

This study illustrates a systematic process of adapting and integrating an evidence-based behavioral approach with an evidence-based biomedical approach. To the best of our knowledge, this is the first study to develop an integrated HIV prevention intervention designed to optimally address PrEP adherence, HIV risk reduction, and cognitive needs of high-risk PWUD in substance abuse treatment.

Consistent with prior findings (23, 53), the FG participants reported that a significant proportion of PWUD in treatment are cognitively impaired. These individuals often present for treatment with deficits in cognitive domains that may negatively affect their treatment outcomes. Importantly, the providers were unaware about the degree of cognitive deficits their patients may have. This has serious implications in terms of designing HIV prevention interventions, suggesting the need to incorporate appropriate strategies and tools to accommodate individuals’ level of cognitive deficits. There were also a number of behavioral and cognitive remediation strategies that were suggested during our FGs and stemming from prior intervention research (54–64). The incorporation of such strategies will help to accommodate clients’ cognitive difficulties, thereby providing the opportunity for all clients to fully engage in and benefit from treatment.

The results of our study also suggest that most participants are unaware of PrEP and are engaging in risky behaviors (e.g., sharing injecting equipment, condomless sex), as is consistent with prior studies (65–69). This provides an evidence that there is significant room for improvement in participants’ level of awareness about PrEP and supports the need for including intervention content targeting these information deficits. When information deficits about PrEP were corrected by describing its potential benefits, interest in PrEP immediately increased. Furthermore, as raised in previous studies, participants in this study were concerned about risk compensation and sexual decision-making, side effects, interaction with methadone, stigma, adherence, and financial costs associated with PrEP (65, 70–73). These have serious implications in terms of designing intervention content. Specifically, there is a need to include PrEP-related information (e.g., effectiveness, side effects, adherence, perceived affordability, risk compensation, effect of continued drug use on PrEP efficacy), develop behavioral skills to adhere to PrEP (e.g., taking it with methadone), and manage side effects. Finally, content should include skills to negotiate PrEP use, means of sustaining motivation, and developing safer sex and drug use practices in the context of PrEP (74).

The results of this study also suggested a need to offering PrEP, along with methadone, at drug treatment clinics (i.e., MMPs). Similar findings have been observed in studies, which have demonstrated that directly observed therapy by MMP providers is cost-effective and efficacious at improving adherence (e.g., ART) and clinical outcomes among PWUD living with HIV (75–78). MMP clients are in contact with service providers on a daily basis for methadone administration, allowing the counselors to monitor clients’ adherence to medication (79). Overall, these findings support a need for the combination approach that comprise both biomedical (i.e., use of PrEP) and behavioral intervention (e.g., motivate clients to adhere to regimen and to modify their risky behaviors or help them to acquire and retain the knowledge and skills necessary for that change) (17–19) for optimizing primary HIV prevention outcomes.

Our formative work of intervention development involved first, adapting an evidence-based behavioral intervention, guided by the ADAPT-ITT model, and then integrating the adapted behavioral intervention with the evidence-based biomedical intervention for implementation among high-risk drug users in treatment. This process was complemented by data elicited through structured FGs with members of the target population and treatment providers. Information gleaned from the study had a significant impact on the features of the resulting biobehavioral HIV prevention approach—now known as CHRP-BB, including the emphasis on certain content, format, and flexibility of intervention delivery. The CHRP-BB intervention is novel in this context in that it includes both behavioral and biomedical components of HIV prevention. Additionally, this combination approach also incorporates certain strategies or tools to accommodate participants with NCI to help them concentrate, learn, and remember details.

The implementation of integrated HIV prevention (i.e., CHRP-BB) requires a comprehensive approach, which involves changes to be made at the organizational level. First, the most important challenge or ethical concern could include concerns about breach of confidentiality during direct observation of PrEP administration. Necessary arrangements (e.g., private room inside a clinic to facilitate supervised dosing and brief counseling) (80) need to be put in place to assure clients that PrEP-related services will not result in a breach of confidentiality. Second, some treatment providers who participated in the FGs, particularly the counselors at the methadone clinic, expressed a lack of expertise regarding PrEP, which could significantly reduce their participation in delivering the integrated PrEP services. One approach could be providing the necessary education and training to MMP providers and counselors, so that they can play an auxiliary role in engaging clients with PrEP use and provide support for treatment adherence, as they would for any other treatment-related services. Furthermore, our results suggest that additional methods such as cell phone reminders (e.g., phone calls, text), pill boxes, calendar marking, or contingency management could be utilized to further encourage adherence to PrEP, beyond what is provided through direct observation of PrEP at the MMP (81–84).

This study has limitations that are inherent to research with a qualitative method (85). We believe, however, that our careful selection of study participants, a well-established analytical approach, and the incorporation of published empirical findings, resulted in a well-informed integrated HIV prevention approach. Small sample size may limit our ability to generalize the findings to a different risk population. Furthermore, we have yet to determine whether our resulting intervention, the CHRP-BB, will result in substantial health-related changes in our target population of high-risk PWUD in drug treatment. This will be studied in an upcoming pilot study to test the feasibility, acceptability, and efficacy of the new combination approach. We will also seek participant feedback following the pilot phase to guide any necessary intervention modifications in preparation for a randomized controlled trial.

## Conclusion

This study details the formative process in preparation to develop an evidence-based biobehavioral approach for HIV prevention—taking into account both published empirical evidence and input from target population and treatment providers—for use with high-risk PWUD in drug treatment. The findings from this study suggest that there is a great need for the combination approach (e.g., biobehavioral intervention) tailored to high-risk PWUD with cognitive impairment. The resulting biobehavioral intervention, CHRP-BB, is designed to address the HIV-related risk behaviors and PrEP uptake and adherence as experienced by many PWUD in treatment. We hope that the process and outcome of this formative research will help to inform similar work in the future as a growing number of EBIs have become widely available, but may not yet be in optimal form for implementation among certain risk populations or within real world clinical settings.

## Ethics Statement

The study protocol was approved by the Institutional Review Board (IRB) at the University of Connecticut and received board approval from APT Foundation Inc. Prior to participating in the study, all individuals were read a standard informed consent document and had an opportunity to have questions answered, and provided written consent for their participation in the FG session. All procedures performed in studies involving human participants were in accordance with the ethical standards of the institutional and/or national research committee and with the 1964 Helsinki Declaration and its later amendments or comparable ethical standards.

## Author Contributions

All the authors contributed substantially to the conception and design of the study. RS and PK contributed to the analysis and interpretation of the data. All the authors contributed to critical review and revision of the manuscript. They have given final approval of the version to be published and agreed to be accountable for all aspects of the work.

## Conflict of Interest Statement

The authors declare that the research was conducted in the absence of any commercial or financial relationships that could be construed as a potential conflict of interest. The reviewer, LM, and handling editor declared their shared affiliation, and the handling editor states that the process nevertheless met the standards of a fair and objective review.

## References

[1] CDC. HIV Surveillance Report: Diagnoses of HIV Infection in the United States and Dependent Areas, 2015. Atlanta, GA: CDC (2016).

[2] Arasteh K Jarlais DCD Perlis TE Alcohol and HIV sexual risk behaviors among injection drug users. Drug Alcohol Depend (2008) 95(1):54–61. 10.1016/j.drugalcdep.2007.12.008 18242009 PMC2373771

[3] Marshall BDL Friedman SR Monteiro JFG Paczkowski M Tempalski B Pouget ER Prevention and treatment produced large decreases in HIV incidence in a model of people who inject drugs. Health Aff (2014) 33(3):401–9. 10.1377/hlthaff.2013.0824 24590937 PMC4469974

[4] Noar SM. Behavioral interventions to reduce HIV-related sexual risk behavior: review and synthesis of meta-analytic evidence. AIDS Behav (2008) 12(3):335–53. 10.1007/s10461-007-9313-9 17896176

[5] Strathdee SA Hallett TB Bobrova N Rhodes T Booth R Abdool R HIV and risk environment for injecting drug users: the past, present, and future. Lancet (2010) 376(9737):268–84. 10.1016/S0140-6736(10)60743-X 20650523 PMC6464374

[6] Volkow ND Montaner J. The urgency of providing comprehensive and integrated treatment for substance abusers with HIV. Health Aff (Millwood) (2011) 30(8):1411–9. 10.1377/hlthaff.2011.0663 21821558 PMC3190599

[7] CDC. Effective Interventions: HIV Prevention That Works. Atlanta, GA: CDC (2016). Available from: https://effectiveinterventions.cdc.gov/

[8] Worley MJ Tate SR Brown SA. Mediational relations between 12-step attendance, depression, and substance use in patients with comorbid substance dependence and major depression. Addiction (2012) 107(11):1974–83. 10.1111/j.1360-0443.2012.03943.x 22578037 PMC3466338

[9] Huedo-Medina TB Shrestha R Copenhaver M. Modeling a theory-based approach to examine the influence of neurocognitive impairment on HIV risk reduction behaviors among drug users in treatment. AIDS Behav (2016) 20(8):1646–57. 10.1007/s10461-016-1394-x 27052845 PMC4945437

[10] Grant RM Lama JR Anderson PL McMahan V Liu AY Vargas L Preexposure chemoprophylaxis for HIV prevention in men who have sex with men. N Engl J Med (2010) 363(27):2587–99. 10.1056/NEJMoa1011205 21091279 PMC3079639

[11] Baeten JM Donnell D Ndase P Mugo NR Campbell JD Wangisi J Antiretroviral prophylaxis for HIV prevention in heterosexual men and women. N Engl J Med (2012) 367(5):399–410. 10.1056/NEJMoa1108524 22784037 PMC3770474

[12] Thigpen MC Kebaabetswe PM Paxton LA Smith DK Rose CE Segolodi TM Antiretroviral preexposure prophylaxis for heterosexual HIV transmission in Botswana. N Engl J Med (2012) 367(5):423–34. 10.1056/NEJMoa1110711 22784038

[13] Van Damme L Corneli A Ahmed K Agot K Lombaard J Kapiga S Preexposure prophylaxis for HIV infection among African women. N Engl J Med (2012) 367(5):411–22. 10.1056/NEJMoa1202614 22784040 PMC3687217

[14] Choopanya K Martin M Suntharasamai P Sangkum U Mock PA Leethochawalit M Antiretroviral prophylaxis for HIV infection in injecting drug users in Bangkok, Thailand (the Bangkok Tenofovir Study): a randomised, double-blind, placebo-controlled phase 3 trial. Lancet (2013) 381(9883):2083–90. 10.1016/S0140-6736(13)61127-7 23769234

[15] CDC. Preexposure Prophylaxis for the Prevention of HIV Infection in the United States—2014: A Clinical Practice Guideline. Washington, DC: Department of Health and Human Services USA, Centers for Disease Control and Prevention (2014).

[16] Kirby T Thornber-Dunwell M Uptake of PrEP for HIV slow among MSM. Lancet (2014) 383(9915):399–400. 10.1016/S0140-6736(14)60137-9 24494225

[17] Bekker L-G Beyrer C Quinn TC Behavioral and biomedical combination strategies for HIV prevention. Cold Spring Harb Perspect Med (2012) 2(8):a007435 10.1101/cshperspect.a007435 22908192 PMC3405825

[18] Brown JL Sales JM DiClemente RJ. Combination HIV prevention interventions: the potential of integrated behavioral and biomedical approaches. Curr HIV/AIDS Rep (2014) 11(4):363–75. 10.1007/s11904-014-0228-6 25216985 PMC4358895

[19] Buchbinder SP Liu A. Pre-exposure prophylaxis and the promise of combination prevention approaches. AIDS Behav (2011) 15(1):72–9. 10.1007/s10461-011-9894-1 21331801 PMC3064892

[20] Meade CS Towe SL Skalski LM Robertson KR. Independent effects of HIV infection and cocaine dependence on neurocognitive impairment in a community sample living in the southern United States. Drug Alcohol Depend (2015) 149:128–35. 10.1016/j.drugalcdep.2015.01.034 25697913 PMC4361251

[21] Potvin S Stavro K Rizkallah E Pelletier J. Cocaine and cognition: a systematic quantitative review. J Addict Med (2014) 8(5):368–76. 10.1097/ADM.0000000000000066 25187977

[22] Anderson AM Higgins MK Ownby RL Waldrop-Valverde D. Changes in neurocognition and adherence over six months in HIV-infected individuals with cocaine or heroin dependence. AIDS Care (2015) 27(3):333–7. 10.1080/09540121.2014.985183 25484035 PMC4305469

[23] Shrestha R Huedo-Medina T Altice F Krishnan A Copenhaver M. Examining the acceptability of mHealth technology in HIV prevention among high-risk drug users in treatment. AIDS Behav (2016). 10.1007/s10461-016-1637-x 28025735 PMC5484759

[24] Shrestha R Karki P Altice F Huedo-Medina T Meyer JP Madden L Correlates of willingness to use pre-exposure prophylaxis (PrEP) and the likelihood of practicing safer drug- and sex-related risk behaviors while on PrEP among high-risk drug users in treatment. Drug Alcohol Depend (2017) 173:107–16. 10.1016/j.drugalcdep.2016.12.023 28214391 PMC5366273

[25] Shrestha R Huedo-Medina T Copenhaver M Sex-related differences in self-reported neurocognitive impairment among high-risk cocaine users in methadone maintenance treatment program. Subst Abuse (2015) 9:17–24. 10.4137/SART.S23332 25861219 PMC4363005

[26] Bates ME Pawlak AP Tonigan JS Buckman JF. Cognitive impairment influences drinking outcome by altering therapeutic mechanisms of change. Psychol Addict Behav (2006) 20(3):241–53. 10.1037/0893-164X.20.3.241 16938062 PMC2965453

[27] Verdejo-Garcia A Perez-Garcia M. Profile of executive deficits in cocaine and heroin polysubstance users: common and differential effects on separate executive components. Psychopharmacology (Berl) (2007) 190(4):517–30. 10.1007/s00213-006-0632-8 17136401

[28] Fishbein DH Krupitsky E Flannery BA Langevin DJ Bobashev G Verbitskaya E Neurocognitive characterizations of Russian heroin addicts without a significant history of other drug use. Drug Alcohol Depend (2007) 90(1):25–38. 10.1016/j.drugalcdep.2007.02.015 17382488 PMC1991277

[29] Vo HT Schacht R Mintzer M Fishman M. Working memory impairment in cannabis- and opioid-dependent adolescents. Subst Abus (2014) 35(4):387–90. 10.1080/08897077.2014.954027 25148203

[30] Anand P Springer S Copenhaver M Altice F. Neurocognitive impairment and HIV risk factors: a reciprocal relationship. AIDS Behav (2010) 14(6):1213–26. 10.1007/s10461-010-9684-1 20232242 PMC2906682

[31] Ezeabogu I Copenhaver MM Potrepka J. The influence of neurocognitive impairment on HIV treatment outcomes among drug-involved people living with HIV/AIDS. AIDS Care (2012) 24(3):386–93. 10.1080/09540121.2011.608794 22250847 PMC3294373

[32] Attonito JM Devieux JG Lerner BD Hospital MM Rosenberg R. Exploring substance use and HIV treatment factors associated with neurocognitive impairment among people living with HIV/AIDS. Front Public Health (2014) 2:105. 10.3389/fpubh.2014.00105 25157345 PMC4127797

[33] Becker BW Thames AD Woo E Castellon SA Hinkin CH. Longitudinal change in cognitive function and medication adherence in HIV-infected adults. AIDS Behav (2011) 15(8):1888–94. 10.1007/s10461-011-9924-z 21437726 PMC3616486

[34] Shrestha R Karki P Huedo-Medina T Copenhaver M Treatment engagement moderates the effect of neurocognitive impairment on antiretroviral therapy adherence in HIV-infected drug users in treatment. J Assoc Nurses AIDS Care (2016) 28(1):85–94. 10.1016/j.jana.2016.09.007 27769735 PMC5183513

[35] O’Connor MK Mueller L Van Ormer A Drake R Penk W Rosenheck R Cognitive impairment as barrier to engagement in vocational services among veterans with severe mental illness. J Rehabil Res Dev (2011) 48(5):597–608. 10.1682/JRRD.2010.06.0117 21674409

[36] Katz EC King SD Schwartz RP Weintraub E Barksdale W Robinson R Cognitive ability as a factor in engagement in drug abuse treatment. Am J Drug Alcohol Abuse (2005) 31(3):359–69. 10.1081/ADA-200056767 16161723

[37] Aharonovich E Nunes E Hasin D. Cognitive impairment, retention and abstinence among cocaine abusers in cognitive-behavioral treatment. Drug Alcohol Depend (2003) 71(2):207–11. 10.1016/S0376-8716(03)00092-9 12927659 PMC5804498

[38] Aharonovich E Hasin DS Brooks AC Liu X Bisaga A Nunes EV. Cognitive deficits predict low treatment retention in cocaine dependent patients. Drug Alcohol Depend (2006) 81(3):313–22. 10.1016/j.drugalcdep.2005.08.003 16171953

[39] Meade CS Conn NA Skalski LM Safren SA. Neurocognitive impairment and medication adherence in HIV patients with and without cocaine dependence. J Behav Med (2011) 34(2):128–38. 10.1007/s10865-010-9293-5 20857187 PMC3049963

[40] Thaler NS Sayegh P Kim MS Castellon SA Hinkin CH. Interactive effects of neurocognitive impairment and substance use on antiretroviral non-adherence in HIV disease. Arch Clin Neuropsychol (2015) 30(2):114–21. 10.1093/arclin/acu092 25589442 PMC4402335

[41] Shrestha R Copenhaver M. The influence of neurocognitive impairment on HIV risk behaviors and intervention outcomes among high-risk substance users: a systematic review. Front Public Health (2016) 4:16. 10.3389/fpubh.2016.00016 26904535 PMC4746254

[42] Shrestha R Weikum D Copenhaver M Altice FL The influence of neurocognitive impairment, depression, and alcohol use disorders on health-related quality of life among incarcerated, HIV-infected, opioid dependent Malaysian men: a moderated mediation analysis. AIDS Behav (2016) 21(4):1070–81. 10.1007/s10461-016-1526-3 PMC5318281 27544515

[43] Wingood GM DiClemente RJ The ADAPT-ITT model: a novel method of adapting evidence-based HIV Interventions. J Acquir Immune Defic Syndr (1999) 2008(47 Suppl 1):S40–6. 10.1097/QAI.0b013e3181605df1 18301133

[44] Wiley TG Literacy and Language Diversity in the United States. Language in Education: Theory and Practice. McHenry, IL:ERIC (1996). 87 p.

[45] Copenhaver M Chowdhury S Altice FL. Adaptation of an evidence-based intervention targeting HIV-infected prisoners transitioning to the community: the process and outcome of formative research for the positive living using safety (PLUS) intervention. AIDS Patient Care STDS (2009) 23(4):277–87. 10.1089/apc.2008.0157 19260773 PMC2770798

[46] Copenhaver MM Tunku N Ezeabogu I Potrepka J Zahari MMA Kamarulzaman A Adapting an evidence-based intervention targeting HIV-infected prisoners in malaysia. AIDS Res Treat (2011) 2011:14. 10.1155/2011/131045 21860786 PMC3157158

[47] Shrestha R Karki P Pandey S Copenhaver M. Adapting an evidence-based HIV prevention intervention targeting high-risk migrant workers: the process and outcome of formative research. Front Public Health (2016) 4:61. 10.3389/fpubh.2016.00061 27066474 PMC4815003

[48] Albrecht TL Johnson GM Walther JB Understanding Communication Processes in Focus Groups. Successful Focus Groups: Advancing the State of the Art. Newbury Park, CA: SAGE Publications (1993). p. 51–64.

[49] Wilkinson S 10 Focus Group Research. Qualitative Research: Theory, Method and Practice. London: SAGE Publications (2004). 177 p.

[50] Dawson S Manderson L Tallo V A Manual for the Use of Focus Groups. Methods for Social Research in Disease. Boston, MA: International Nutrition Foundation for Developing Countries (1993).

[51] Dowling M; ATLAS.ti (Software). The SAGE Encyclopedia of Qualitative Research Methods. Thousand Oaks, CA: SAGE (2008). p. 37–8.

[52] Copenhaver M Lee I Baldwin P. A randomized controlled trial of the community-friendly health recovery program (CHRP) among high-risk drug users in treatment. AIDS Behav (2013) 17(9):2902–13. 10.1007/s10461-013-0539-4 23835735 PMC3878981

[53] Schouten J Cinque P Gisslen M Reiss P Portegies P. HIV-1 infection and cognitive impairment in the cART era: a review. AIDS (2011) 25(5):561–75. 10.1097/QAD.0b013e3283437f9a 21160410

[54] McDonell MG Srebnik D Angelo F McPherson S Lowe JM Sugar A Randomized controlled trial of contingency management for stimulant use in community mental health patients with serious mental illness. Am J Psychiatry (2013) 170(1):94–101. 10.1176/appi.ajp.2012.11121831 23138961 PMC4242089

[55] Park K-M Ku J Choi S-H Jang H-J Park J-Y Kim SI A virtual reality application in role-plays of social skills training for schizophrenia: a randomized, controlled trial. Psychiatry Res (2011) 189(2):166–72. 10.1016/j.psychres.2011.04.003 21529970

[56] Bellack AS Brown CH Thomas-Lohrman S. Psychometric characteristics of role-play assessments of social skill in schizophrenia. Behav Ther (2006) 37(4):339–52. 10.1016/j.beth.2006.01.005 17071212

[57] Kurtz MM Mueser KT A meta-analysis of controlled research on social skills training for schizophrenia. J Consult Clin Psychol (2008) 76(3):491–504. 10.1037/0022-006X.76.3.491 18540742

[58] Bauer S Okon E Meermann R Kordy H. Technology-enhanced maintenance of treatment gains in eating disorders: efficacy of an intervention delivered via text messaging. J Consult Clin Psychol (2012) 80(4):700–6. 10.1037/a0028030 22545736

[59] Harvey AG Lee J Williams J Hollon SD Walker MP Thompson MA Improving outcome of psychosocial treatments by enhancing memory and learning. Perspect Psychol Sci (2014) 9(2):161–79. 10.1177/1745691614521781 25544856 PMC4276345

[60] Copenhaver M Avants SK Warburton LA Margolin A. Intervening effectively with drug abusers infected with HIV: taking into account the potential for cognitive impairment. J Psychoactive Drugs (2003) 35(2):209–18. 10.1080/02791072.2003.10400002 12924743

[61] Epstein ML Lazarus AD Calvano TB Matthews KA Hendel RA Epstein BB Immediate feedback assessment technique promotes learning and corrects inaccurate first responses. Psychol Rec (2002) 52(2):187–201.

[62] Knouse LE Safren SA. Current status of cognitive behavioral therapy for adult attention-deficit hyperactivity disorder. Psychiatr Clin North Am (2010) 33(3):497–509. 10.1016/j.psc.2010.04.001 20599129 PMC2909688

[63] Cooper L Combined motivational interviewing and cognitive-behavioral therapy with older adult drug and alcohol abusers. Health Soc Work (2012) 37(3):173–9. 10.1093/hsw/hls023 23193732

[64] Insel KC Einstein GO Morrow DG Hepworth JT. A multifaceted prospective memory intervention to improve medication adherence: design of a randomized control trial. Contemp Clin Trials (2013) 34(1):45–52. 10.1016/j.cct.2012.09.005 23010608 PMC4407997

[65] Auerbach JD Kinsky S Brown G Charles V. Knowledge, attitudes, and likelihood of pre-exposure prophylaxis (PrEP) use among US women at risk of acquiring HIV. AIDS Patient Care STDS (2015) 29(2):102–10. 10.1089/apc.2014.0142 25513954 PMC4321978

[66] Al-Tayyib AA Thrun MW Haukoos JS Walls NE. Knowledge of pre-exposure prophylaxis (PrEP) for HIV prevention among men who have sex with men in Denver, Colorado. AIDS Behav (2014) 3:340–7. 10.1007/s10461-013-0553-6 23824227 PMC4066993

[67] Mehta SA Silvera R Bernstein K Holzman RS Aberg JA Daskalakis DC. Awareness of post-exposure HIV prophylaxis in high-risk men who have sex with men in New York City. Sex Transm Infect (2011) 87(4):344–8. 10.1136/sti.2010.046284 21357600

[68] CDC. HIV infection and HIV-associated behaviors among injecting drug users – 20 cities, United States, 2009. MMWR Morb Mortal Wkly Rep (2012) 61(8):133–8. 22377843

[69] Linton SL Celentano DD Kirk GD Mehta SH. The longitudinal association between homelessness, injection drug use, and injection-related risk behavior among persons with a history of injection drug use in Baltimore, MD. Drug Alcohol Depend (2013) 132(3):457–65. 10.1016/j.drugalcdep.2013.03.009 23578590 PMC3926693

[70] Brooks RA Landovitz RJ Kaplan RL Lieber E Lee S-J Barkley TW Sexual risk behaviors and acceptability of HIV pre-exposure prophylaxis among HIV-negative gay and bisexual men in serodiscordant relationships: a mixed methods study. AIDS Patient Care STDS (2011) 26(2):87–94. 10.1089/apc.2011.0283 22149764 PMC3266517

[71] Golub SA Kowalczyk W Weinberger CL Parsons JT. Preexposure prophylaxis and predicted condom use among high-risk men who have sex with men. J Acquir Immune Defic Syndr (2010) 54(5):548–55. 10.1097/QAI.0b013e3181e19a54 20512046 PMC2908204

[72] Liu A Cohen S Follansbee S Cohan D Weber S Sachdev D Early experiences implementing pre-exposure prophylaxis (PrEP) for HIV prevention in San Francisco. PLoS Med (2014) 11(3):e1001613 10.1371/journal.pmed.1001613 24595035 PMC3942317

[73] Puro V Palummieri A De Carli G Piselli P Ippolito G. Attitude towards antiretroviral pre-exposure prophylaxis (PrEP) prescription among HIV specialists. BMC Infect Dis (2013) 13(1):217. 10.1186/1471-2334-13-217 23672424 PMC3658955

[74] Shrestha R Altice FL Huedo-Medina TB Karki P Copenhaver M Willingness to use pre-exposure prophylaxis (PrEP): an empirical test of the information-motivation-behavioral skills (IMB) model among high-risk drug users in treatment. AIDS Behav (2016) 21(5):1299–308. 10.1007/s10461-016-1650-0 PMC5380590 27990587

[75] Brust JC Litwin AH Berg KM Li X Heo M Arnsten JH. Directly observed antiretroviral therapy in substance abusers receiving methadone maintenance therapy does not cause increased drug resistance. AIDS Res Hum Retroviruses (2011) 27(5):535–41. 10.1089/aid.2010.0181 20854173 PMC3083727

[76] Nahvi S Litwin AH Heo M Berg KM Li X Arnsten JH. Directly observed antiretroviral therapy eliminates adverse effects of active drug use on adherence. Drug Alcohol Depend (2012) 120(1–3):174–80. 10.1016/j.drugalcdep.2011.07.025 21885212 PMC3245772

[77] Uhlmann S Milloy MJ Kerr T Zhang R Guillemi S Marsh D Methadone maintenance therapy promotes initiation of antiretroviral therapy among injection drug users. Addiction (2010) 105(5):907–13. 10.1111/j.1360-0443.2010.02905.x 20331553 PMC2857602

[78] Berg KM Litwin AH Li X Heo M Arnsten JH. Lack of sustained improvement in adherence or viral load following a directly observed antiretroviral therapy intervention. Clin Infect Dis (2011) 53(9):936–43. 10.1093/cid/cir537 21890753 PMC3189166

[79] Achmad YM Istiqomah AN Iskandar S Wisaksana R van Crevel R Hidayat T. Integration of methadone maintenance treatment and HIV care for injecting drug users: a cohort study in Bandung, Indonesia. Acta Med Indones (2009) 41(Suppl 1):23–7. 19920294

[80] Lin C Cao X Li L. Integrating antiretroviral therapy in methadone maintenance therapy clinics: service provider perceptions. Int J Drug Policy (2014) 25(6):1066–70. 10.1016/j.drugpo.2014.04.021 24939555 PMC4226744

[81] Pop-Eleches C Thirumurthy H Habyarimana JP Zivin JG Goldstein MP De Walque D Mobile phone technologies improve adherence to antiretroviral treatment in a resource-limited setting: a randomized controlled trial of text message reminders. AIDS (2011) 25(6):825 10.1097/QAD.0b013e32834380c1 21252632 PMC3718389

[82] Hardy H Kumar V Doros G Farmer E Drainoni M-L Rybin D Randomized controlled trial of a personalized cellular phone reminder system to enhance adherence to antiretroviral therapy. AIDS Patient Care STDS (2011) 25(3):153–61. 10.1089/apc.2010.0006 21323532 PMC3101947

[83] Miller CW Himelhoch S Acceptability of mobile phone technology for medication adherence interventions among HIV-positive patients at an urban clinic. AIDS Res Treat (2013) 2013:670525 10.1155/2013/670525 23997948 PMC3755447

[84] Belzer ME Naar-King S Olson J Sarr M Thornton S Kahana SY The use of cell phone support for non-adherent HIV-infected youth and young adults: an initial randomized and controlled intervention trial. AIDS Behav (2014) 18(4):686–96. 10.1007/s10461-013-0661-3 24271347 PMC3962719

[85] Taylor SJ Bogdan R DeVault M Introduction to Qualitative Research Methods: A Guidebook and Resource. Hoboken, NJ:John Wiley & Sons (2015).

